# Dual atrioventricular nodal non-reentrant tachycardia: an often overlooked diagnosis

**DOI:** 10.1093/ehjcr/ytae064

**Published:** 2024-02-08

**Authors:** Bernard Belhassen

**Affiliations:** Hadassah Medical Center, Heart Institute, Kalman Ya'Akov Man Street, Jerusalem 91120, Israel

**This commentary refers to the article ‘Interpolated Junctional Extrasystoles Mimicking Complex Polymorphic Ventricular Arrhythmias in a Healthy Young Athlete: A Case Report.’ by N. Martini *et al*. https://doi.org/10.1093/ehjcr/ytae012.**

Nicolò Bortoli and co-workers recently described the case of a healthy young athlete with recurrent asymptomatic burst of tachycardia associated with both narrow and wide QRS complexes.^1^

The arrhythmia was first interpreted as polymorphic ventricular tachycardia that led to temporary sports ineligibility. However, the authors suggested that the short bursts of tachycardia were related to interpolated junctional ectopics having various degree of ventricular aberration, and thus enabled the athlete to return to normal sportive activities.

**Figure 1:**
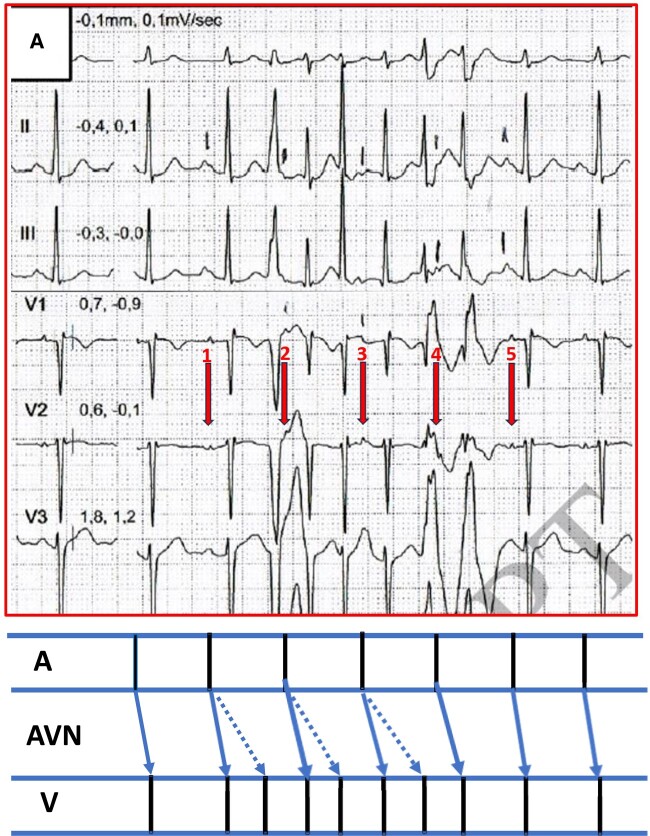
Upper panel: modified figure from the original paper by Martini *et al*.^1^ Lower panel: diagram of the above ECG above. The red arrows represent the normal sinus atrial activity. The blue filled and dashed arrows represent the fast and slow AV nodal pathways, respectively.

I believe that a better and simpler explanation of the case findings involves the simultaneous antegrade conduction of a sinus beat over both a fast atrioventricular (AV) nodal pathway and a slow AV nodal pathway (*Figure 1*). In this instance, the QRS complexes with a left or right bundle branch block pattern reflect the aberrancy resulting from the early capture of the ventricle during conduction over the slow pathway.

Dual AV nodal non-reentrant tachycardia is a rare type of supraventricular tachycardia^2,3^ that is frequently misdiagnosed, especially as atrial fibrillation and less commonly as ventricular tachycardia.^3^ Its diagnosis is usually easily to ascertain during electrophysiologic study and to cure with simple radiofrequency ablation of the slow pathway. In difficult cases, specific pacing methods have been shown to consistently and reproducibly unmask the existence of the dual AV nodal pathways.^4^
